# Relevance and consequence of chronic inflammation for obesity development

**DOI:** 10.1186/s40348-023-00170-6

**Published:** 2023-11-14

**Authors:** Lisa Ruck, Susanna Wiegand, Peter Kühnen

**Affiliations:** 1 Klinik Für Pädiatrische Endokrinologie und Diabetologie, Charité Universitätsmedizin, Berlin, Germany; 2 Berlin Institute of Health at Charité–Universitätsmedizin Berlin, BIH Biomedical Innovation Academy, BIH Charité Junior Clinician Scientist Program, Charitéplatz 1, 10117 Berlin, Germany; 3 Abteilung Interdisziplinär, Sozial-Pädiatrisches Zentrum, Charité Universitätsmedizin, Berlin, Germany

**Keywords:** Inflammation, Adipose tissue, Leptin-melanocortin signaling pathway, Hypothalamus, Incretins, NASH, NAFLD

## Abstract

**Background:**

Increasing prevalence of morbid obesity accompanied by comorbidities like type 2 diabetes mellitus (T2DM) led to a demand for improving therapeutic strategies and pharmacological intervention options. Apart from genetics, inflammation processes have been hypothesized to be of importance for the development of obesity and related aspects like insulin resistance.

**Main text:**

Within this review, we provide an overview of the intricate interplay between chronic inflammation of the adipose tissue and the hypothalamus and the development of obesity. Further understanding of this relationship might improve the understanding of the underlying mechanism and may be of relevance for the establishment of new treatment strategies.

## Background

The overwhelming increase in obesity and its associated comorbidities worldwide necessitates an advancement of optimal therapeutic intervention. However, an understanding of the underlying mechanism is relevant in order to development new strategies to optimize patient management which includes the reduction of obesity-related comorbidities. Special attention has been paid to elucidating the relationship between chronic inflammation and obesity. In contrast to the transient, acute inflammation type, which is characterized by edema formation and leukocyte migration, chronic inflammation endures over a prolonged period and is marked by the presence of lymphocytes and macrophages, which are integral components of adipose tissue [1]. Chronic inflammation has been extensively studied as a component of the metabolic syndrome due to the release of pro-inflammatory adipokines, such as leptin, interleukin-6 (IL-6), tumor necrosis factor-α (TNFα), and others, by adipose tissue [2]. This chronic inflammatory state plays a pivotal role in the pathogenesis of various conditions including fatty liver disease, cardiovascular disease, insulin resistance in T2DM, asthma, neurodegeneration, certain cancers, and predisposition to autoimmune diseases [3, 4]. The presence of pro-inflammatory cytokines and the recruitment of myeloid cells have been shown to directly correlate with metabolic dysfunction observed in obese patients [5–8]. Additionally, obesity-related insulin resistance can impact the adaptive immune response [9, 10]. Impaired T cell function has been observed in mice with diet-induced obesity, leading to poorer outcomes in viral infections such as influenza [11, 12]. The inflammasome, a macromolecular sensor found in innate immune cells, represents a critical initiator of the inflammatory response. This multimeric protein complex is activated by cellular nutrients such as glucose or free fatty acids, exerting control over IL-1β production and Caspase-1 activation among others [13, 14]. The concept of “immune metabolism” encompasses the intricate interplay between immunological processes and metabolic abnormalities. This review aims to provide a detailed summary of the interconnections between inflammation in adipose tissue, the hypothalamus, and the leptin-melanocortin signaling pathway and its pharmacological relevance.

## Main text

### Inflammation and leptin-melanocortin signaling pathway

The leptin-melanocortin signaling pathway plays a crucial role in central appetite regulation. The hormone leptin (LEP), produced by and according to adipose tissue mass, binds to the Leptin receptor (LEPR) in the hypothalamus. This binding stimulates the production of pro-opiomelanocortin (POMC), which is subsequently processed into α-melanocyte-stimulating hormone (MSH) and β-MSH, among other peptides. α- and β-MSH bind to the Melanocortin-4 receptor (MC4R), thereby activating the feeling of satiety, which leads to a reduction in food intake and a modulated energy expenditure [15]. Genetic alterations within this signaling pathway, such as in the *LEP* and *LEPR* gene, lead to severe early-onset adiposity due to hyperphagia [16].

In addition to its role in appetite regulation, leptin also triggers proliferative signals in hematopoiesis and lymphopoiesis. It can activate neutrophils, natural killer cells, monocytes, dendritic cells, and macrophages [17–21]. Additionally, there is an enhanced expression of *leptin* mRNA and cytokines such as TNFα, IL-6, and IL-1β in response to lipopolysaccharide (LPS) stimulation, indicating its role as a mediator in inflammatory activity [22, 23]. In the absence of leptin, dendritic cells exhibit a T helper cell type 2 (Th2)-biased cytokine profile whereas exogenous administration of leptin drives the balance towards a Th1 profile [17, 18]. Th1 responses are present in autoimmune processes, thus reduced levels of Leptin have a protective effect in autoimmune diseases [24–27]. During acute inflammatory reactions and sepsis, a marked increase in leptin levels is observed in the blood of so far healthy individuals. Leptin acts via binding to LEPR, a class 1 cytokine receptor of the superfamily [28–31], which is mainly expressed in the hypothalamus, but also in the kidney, lung, and choroid plexus [32].

Both Leptin-deficient *ob/ob* mice and Leptin receptor-deficient *db/db* mice display impaired cell-mediated immunity and lymphoid atrophy, making them more susceptible to infections and injuries [33–37]. These animals also exhibit thymic atrophy, which affects the maturation process of thymocytes that require leptin as a survival factor [33]. Consequently, specific alterations in peripheral T cell populations can be observed in these animals. Short-term administration of leptin can restore thymic cellularity, reverse LPS-induced thymic atrophy, and support thymopoiesis [38, 39]. On the other hand, *ob/ob* mice appear to be partially protected against inflammation and tissue damage, such as in fulminant hepatitis [40]. They are also resistant to dextran sulfate sodium (DSS)-induced colitis [40] and autoimmune glomerulonephritis [41]. *Lepr*-deficient mice also display impaired lymphopoiesis with reduced numbers of B cells in the bone marrow and permanently reduced levels of B cells and CD4 + T cells in the blood [42]. Hence, a direct role in the proliferation and expansion of hematopoietic stem cells and lymphoid progenitor cells is postulated. Additionally, the development of natural killer (NK) cells is affected, with significantly reduced NK pool size [21].

Patients deficient in *LEP* and *LEPR* show reduced lymphocyte proliferation and cytokine production, making them more prone to infections. Particularly in individuals with Leptin deficiency, an increased incidence of infection-related deaths during childhood has been observed [27, 43]. However, the administration of Leptin can restore these immunological abnormalities [26]. Additionally, low levels of Leptin can also play a crucial role in immunosuppression during periods of starvation and malnutrition [44]. On the other hand, in patients with active rheumatoid arthritis, an inverse correlation between disease activity/inflammation and blood leptin concentration has been observed [45].

Several decades ago, it was demonstrated that α-MSH can downregulate pro-inflammatory cytokines, including IL-1, IL-6, TNFα, as well as immunomodulatory cytokines such as IL-2, IL-4, IL-13, and interferon-γ (INFγ) in vitro [46]. Moreover, cell experiments have revealed that α-MSH influences the production of immunoglobulin E (IgE) and nitric oxide (NO) and inhibits IL-1β-induced production of IL-8, growth-regulated protein α (Groα), and nuclear factor “kappa-light-chain-enhancer” of activated B cells (NFκB) [47, 48]. In mouse models, administration of α-MSH suppressed allergic airway inflammation and reduced levels of Il-4 and Il-13 in the bronchoalveolar lavage of allergic mice [49]. Similarly, in mice with DSS-induced colitis, α-MSH administration mitigated disease-induced weight loss and improved the overall outcome of the animals [50]. These effects are believed to be mediated through the melanocortin-1 receptor (MC1R). MC1R, primarily known for its role in melanocyte pigmentation [51, 52], is also expressed in immune cells [53–55]. Concordantly, Mc1r-deficient mice exhibited significantly worse outcomes in DSS-induced colitis, characterized by increased weight loss and more pronounced histological changes compared to wild-type mice [56]. Thus, it can be postulated that MC1R serves as an important regulator of mucosal defense. Mutations in *MC1R* lead to an augmented inflammatory response and are associated with burn-induced systemic inflammatory response syndrome (SIRS) and infectious complications in patients [57, 58]. Furthermore, MC1R is implicated in the development of hypertrophic scarring [59]. Studies have shown that administration of an MC1R agonist (PL-8177) significantly reduced the inflammatory response in mice with experimentally-induced autoimmune uveitis [60] and experimentally-induced inflammatory bowel disease in rats [61]. The MC4R agonist setmelanotide (RM493), which is approved by the U.S. Food and Drug Administration (FDA) and the European Medicines Agency (EMA) for the treatment of monogenic obesity in LEPR- and POMC-deficient patients, also binds to MC1R, resulting in skin hyperpigmentation and hair darkening in patients [62–64]. In vitro experiments demonstrated that activation of MC4R by setmelanotide in astrocytes exhibits anti-inflammatory and neuroprotective effects. Astrocytoma cells incubated with TNFα and IFNγ and subsequently treated with setmelanotide exhibited reduced expression of chemokine C–C motif ligand 2 (CCL2) and C-X-C motif chemokine 10 (CXCL10), while *IL-6* and *IL-11* mRNA levels were increased. These chemokines play an important role in the activation of leukocytes in the central nervous system (CNS) [65].

**Fig. 1:**
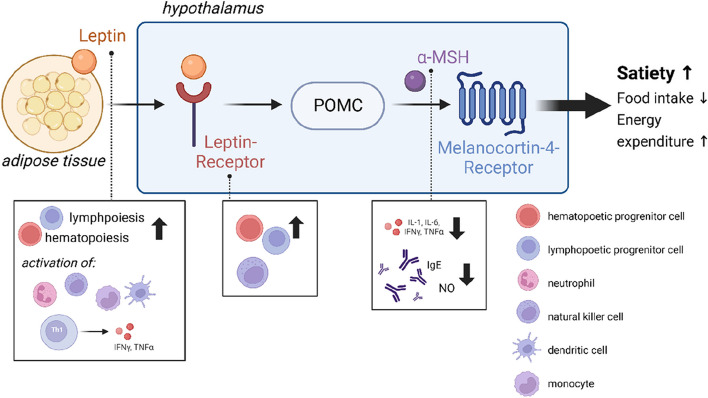
Components of the leptin-melanocortin signaling pathway contribute to immunological functions. Leptin is produced by the adipose tissue and binds centrally to the leptin-receptor. This activates the production and processing of POMC into α-MSH among others. Α-MSH binds to the MC4R, which initiates a feeling of satiety and leading to a reduction and food intake. The components of this pathway also contribute to inflammatory functions. Leptin acts via binding to the Leptin-receptor and increases hematopoiesis and lymphopoiesis. It activates neutrophils, NK, monocytes, dendritic cells, and macrophages and promotes a Th1-type production of pro-inflammatory cytokines. Α-MSH has been shown to downregulate pro-inflammatory cytokines such as IL-1, IL-6, and TNFα and immunomodulatory cytokines like IL-2, IL-4, IL-13, and INFγ. It also reduces the production of IgE and NO

In addition to its role in regulating hunger and satiety, the individual components of the leptin-melanocortin signaling pathway also contribute to immunomodulatory responses. Leptin conveys pro-inflammatory signals via activation of the immune system while, antagonistically, α-MSH displays anti-inflammatory effects for example in inflammatory bowel diseases (Fig. 1).

### Inflammation and adipose tissue

**Fig. 2:**
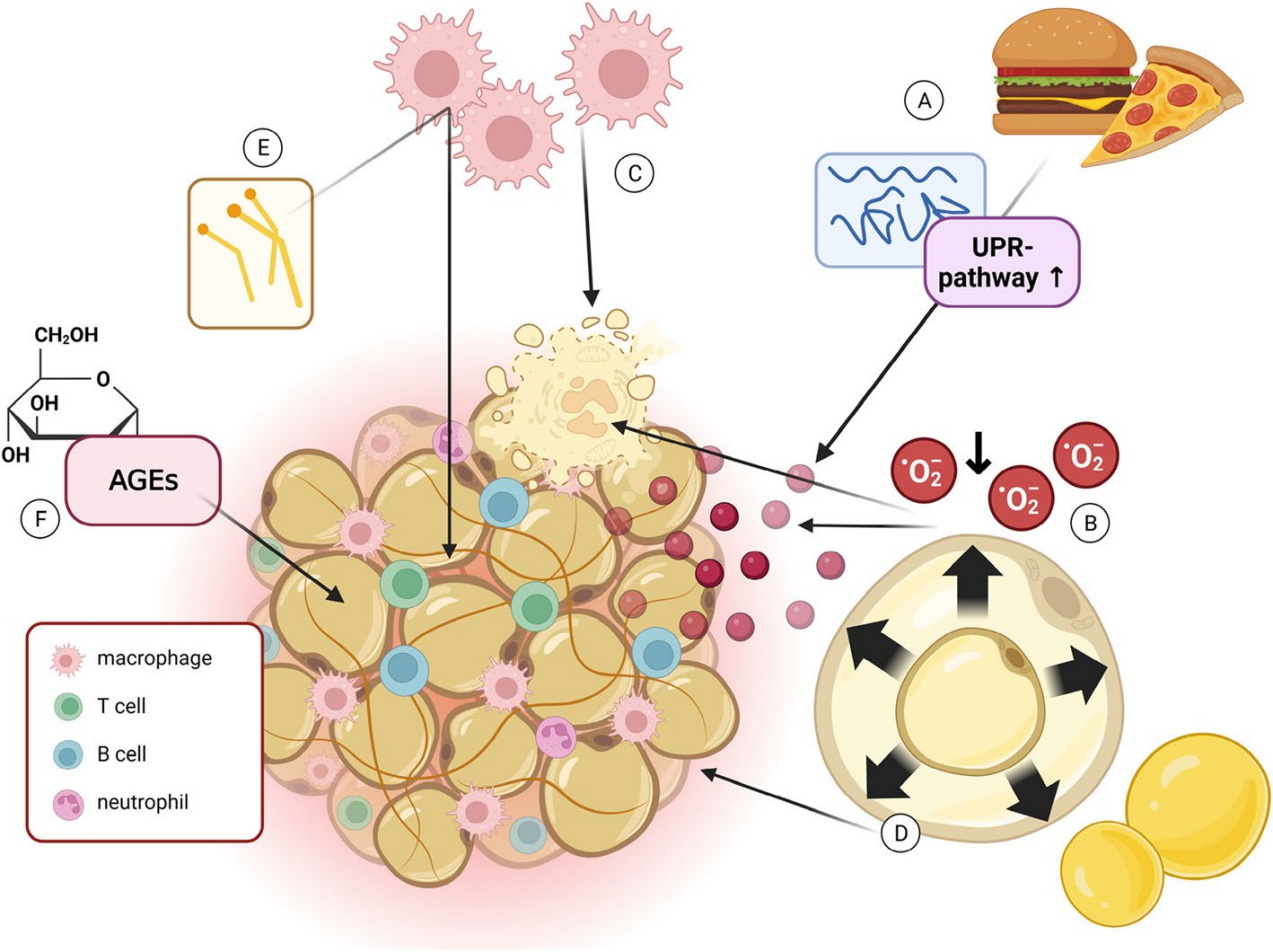
Activation of chronic inflammation in the adipose tissue. Several hypotheses suggest possible mechanisms for the activation of adipose tissue inflammation. Hypothesis (**a**) proposes that an increased nutrient intake leads to an accumulation of misfolded/unfolded proteins, which activates the UPR, activating inflammation. Hypothesis (**b**) postulates that overloading of adipocytes triggers infiltration of macrophages due to hypoxia, followed by activation of cytotoxic T cells, which subsequently initiate inflammatory cascades. Hypoxia results in necrosis, which further promotes macrophage infiltration (see **c**). Overloaded adipocytes and consequent mechanical stress can also directly activate immune pathogen sensors (**d**). Lastly, free fatty acids can promote inflammation via indirectly binding to TLRs, which activates JKN1. This in turn stimulates the secretion of chemokines, such as MCP1 (**e**). AGEs are also postulated to contribute to inflammation in the adipose tissue (**f**)

Multiple hypotheses have been proposed to elucidate the mechanisms underlying chronic adipose tissue inflammation in obesity (Fig. 2). The first hypothesis suggests that excessive nutrient intake leads to the accumulation of misfolded or unfolded proteins in the endoplasmic reticulum, triggering the activation of the unfolded protein response pathway (UPR), which leads to an enhanced expression of pro-inflammatory cytokines [66–71]. The second hypothesis postulates that an overloading of adipocytes leads to a substantial infiltration of macrophages. This is accompanied by the differentiation and activation of cytotoxic T cells, which subsequently initiate inflammatory cascades [72–74]. Another theory focuses on the expansion of adipose tissue, which results in reduced perfusion, leading to hypoxia and subsequent activation of pro-inflammatory signaling pathways. This hypoxia-induced inflammation contributes to necrosis and infiltration of macrophages within the adipose tissue [75–78]. Moreover, overloaded adipocytes and mechanical stress directly activate immune pathogen sensors, further promoting chronic inflammation [79]. Additionally, free fatty acids in the adipose tissue can promote inflammation by indirectly binding to Toll-like receptors (TLR4 and TLR2), leading to the activation of NFκB and Janus kinase 1 (JNK1) [80, 81]. This activation, in turn, stimulates the synthesis and secretion of chemokines, such as monocyte chemoattractant protein-1 (MCP1), by adipocytes and macrophages. These chemokines contribute to the infiltration of pro-inflammatory macrophages [82, 83]. As a result, this local adipose tissue inflammation triggers systemic inflammation, which is closely associated with the development of obesity-related comorbidities. This also includes inflammatory vascular changes, which can finally lead to atherosclerotic cardiovascular diseases (CVD). In patients with CVD, plasma adiponectin levels are decreased [84]. Adiponectin is proposed to be protective against CVD by repressing inflammatory mediators such as vascular cell adhesion molecule 1 (VCAM1), TNFα, and IL-6 and by stimulating endothelial NO synthase [85–88]. Therefore, adipokines are suggested to play an important role in CVD. Furthermore, it has been postulated that advanced glycation end products (AGEs) may play a contributory role in adipose tissue inflammation. AGEs, comprising proteins and lipids subjected to glycation by various sugars, most notably glucose, exhibit their function by binding to cell surfaces or receptors and by catalyzing ROS formation and accumulation [89]. Notably, AGE levels are increased in patients with hyperglycemia, which can activate different signaling pathways, including NF-kB, which regulates the transcription of proteins, such as chemokines, growth factors, or cytokines [90].

**Fig. 3:**
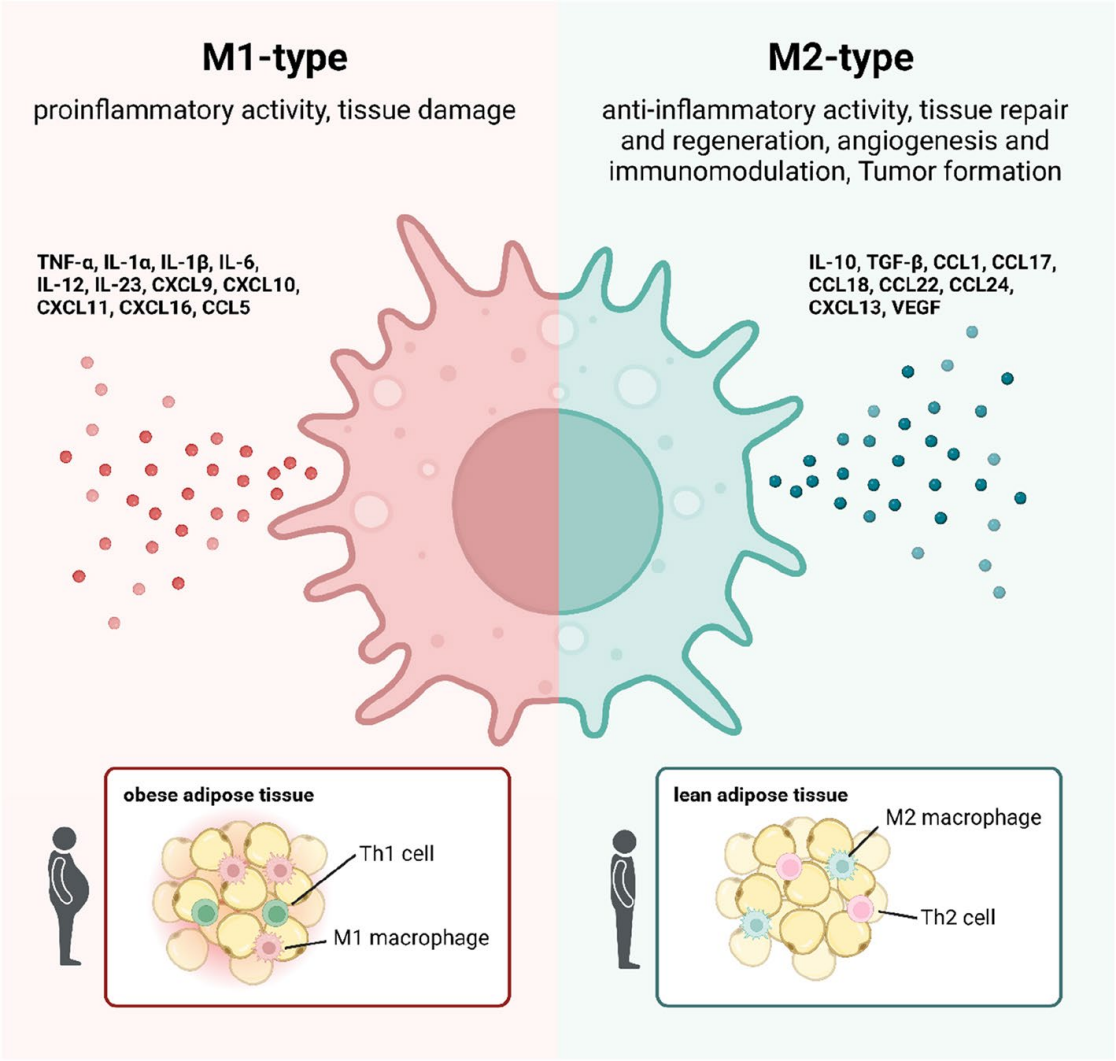
M1-type macrophages are prevalent in obese adipose tissue. In obese adipose tissue, pro-inflammatory M1-type macrophages are predominantly activated. They mainly produce cytokines and chemokines with inflammatory activity such as TNFα, IL-1α, IL-1β, and IL-6. Additionally, Th1 cells are mainly infiltrating the obese adipose tissue. In lean adipose tissue, the anti-inflammatory M2-type macrophage is dominantly represented. These macrophages mainly participate in tissue repair and regeneration and produce cytokines like IL-10 and transforming growth factor β (TGFβ). Apart from M2 macrophages, also Th2 cells are localized in the lean adipose tissue

Our adipose tissue depot harbors approximately 2–5 million cells per gram, of which around 65% are leukocytes. Consequently, adipose tissue functions as an autonomous immunological organ [5–8]. Within our visceral adipose tissue, a diverse array of immune cells exists, including macrophages, dendritic cells, granulocytes, lymphocytes, T cells, and B cells [91]. Remarkably, up to 15 distinct subpopulations of leukocytes can be discerned [92]. In contrast, subcutaneous fat is prominent in lean subjects, and rather serves as a barrier against dermal infection and physical external stress as well as an important regulator of body temperature and is therefore much less immunologically active [93]. Neutrophils are the initial immune cells to infiltrate the visceral adipose tissue in obesity, initiating inflammation within the adipose depot [94]. Macrophages constitute approximately 4% of the healthy visceral adipose tissue, which can escalate to 12% in the context of obesity [83]. Two distinguishable macrophage populations are present: M1 (type 1 macrophage), prevalent in obesity, and M2, predominantly found in lean adipose tissue [95] (Fig. 3). M1 macrophages exhibit an increased production of pro-inflammatory cytokines, such as IL-6, TNFα, IL-12, and IL-23, alongside a reduced synthesis of the anti-inflammatory cytokine IL-10 [95]. M2 macrophages primarily engage in tissue repair processes and generate IL-10, concomitant with decreased IL-12 and IL-23 synthesis [96]. Studies with high-fat-diet (HFD)-induced obesity mouse models have demonstrated a significant increase in NK cells, responsible for the M1 polarization of macrophages through IFNγ production [97, 98]. B cells also exhibit heightened abundance within the adipose tissue of obese individuals [99]. Preventing the accumulation of adipose tissue macrophages (ATMs) or pro-inflammatory macrophages holds the potential to shield obese mice from glucose intolerance and insulin resistance [100–102]. Consistently, the reduction of B cells in obesity culminates in enhanced insulin sensitivit [6]. Mice incapable of producing inflammasome molecules exhibit improved glucose tolerance and insulin sensitivity when subjected to HFD compared to their wild-type counterparts [103–107]. Administering obese mice with a caspase-1 inhibitor therapy can restore metabolic functions. Consequently, these inhibitors present a therapeutic potential for inflammasome-targeted interventions [104].

Pharmaceuticals targeting immunological modulators have been shown to improve insulin sensitivity in humans as well. For example, in patients afflicted with both rheumatoid arthritis and diabetes, IL-1R antagonist has demonstrated the capacity to enhance insulin sensitivity [108]. Clinical trials have unveiled the potential of TNFα antagonists in inhibiting the development of type 2 diabetes [109, 110]. These antagonists have exhibited improvements in glycemic control among obese patients with psoriasis, rheumatoid arthritis, and Crohn's disease who do not have diabetes [111–118]. Additionally, Anakinra, a recombinant human IL-1R antagonist, has been found to ameliorate the secretory function of B cells and reduce glycemic levels [119]. Ongoing clinical trials are presently investigating the effects of neutralizing anti-IL-1β antibodies, particularly in patients with type 2 diabetes [108, 120–122].

On the other hand, there is also therapeutic potential focusing on anti-inflammatory adipokines produced by the adipose tissue, such as adiponectin, to prevent the incidence of co-morbidities like insulin resistance [123–127]. Adiponectin exerts beneficial effects on inflammation, atherosclerosis [128], T2DM, and insulin resistance [129, 130]. It enhances local NO production [88], protects against endothelial dysfunction, and inhibits plaque formation and thrombosis. Consequently, it serves as a vasoprotective factor while mitigating oxidative stress [131, 132]. It also improves insulin sensitivity, impedes the uptake of non-esterified fatty acids, reduces gluconeogenesis, and augments oxidative processes. Consistently, in cases of severe weight gain and obesity, adiponectin levels are notably diminished [133, 134].

Our microbiome also exerts a significant influence on the immune response and could serve as a prospective therapeutic target in managing insulin resistance and chronic inflammation associated with obesity [135]. A research group has demonstrated that patients who undergo Roux-en-Y gastric bypass surgery display diminished infiltration of macrophages in adipose tissue, resulting in reduced inflammation [136].

Inflammation within the adipose tissue has been a long-known regulator of the development of metabolic syndrome. In recent years, targeting this chronic state of inflammation has led to the development of new pharmacological strategies to protect against insulin resistance and diet-induced obesity (DIO), indicating that a more extensive understanding of underlying mechanisms can contribute to improved therapy strategies for patients with obesity and metabolic syndrome.

### Inflammation and hypothalamus

Gut inflammation is a consequential outcome of an HFD and potentially contributes to the onset of obesity [137, 138]. The intricate interplay between dietary components of HFD, the microbiome, and neuronal inflammation holds substantial importance [139]. HFD induces noteworthy alterations in the diversity of the microbiome and triggers oxidative stress within the hypothalamus [140]. As a consequence, the permeability of the blood–brain barrier is enhanced due to a potential downregulation of tight junction proteins [141–143]. This enables the infiltration of peripheral macrophages into the hypothalamus [144, 145]. These infiltrating macrophages originate from adipose tissue and share similar surface markers with ATMs. Displaying a pro-inflammatory M1 phenotype may contribute to neuropathological conditions such as cerebral ischemia and dementia [146].

In male mice, an HFD leads to a rise in macrophage population from 1.3 to 2.9% of all hypothalamic cells. Concurrently, the proportion of macrophages in visceral adipose tissue also increases from 5.3% to as high as 22.8% in these animals. Furthermore, the proportion of microglia cells in the hypothalamus of male mice increases from 31 to 52% following HFD [146]. Hypothalamic microglial cells are believed to have an orchestrating role in the inflammatory response as sensors within the hypothalamus [147]. Simultaneously, an increase in the population of these cells has been associated with neurodegeneration [148]. Microglial cells, known as the brain's macrophages, play a crucial role in hypothalamic inflammation [149, 150]. The proportion of microglial cells also influences the strength of the inflammatory response, impacts neuronal stress, and regulates satiety-signaling neurons [151]. This inflammatory response triggers reactive gliosis, characterized by increased infiltration of microglia and proliferation of astrocytes [139, 152]. Among the regulators of microglial cell activation, uncoupling protein 2 (UCP2) plays a significant role. HFD induces mitochondrial changes in microglial cells through an increase in UCP2, leading to the production of reactive oxygen species (ROS) and activation of inflammation [153, 154]. It is therefore highly expressed in activated microglia cells [155]. Concordantly, genetic ablation of UCP2 in microglia cells of mice led to protection against DIO and made POMC-neurons more sensible towards glucose [156]. UCP2 can inhibit the activation of POMC neurons induced by glucose while activating NPY/AgRP neurons through ROS [157] thereby promoting orexigenic signaling. It is distributed throughout the organism including the spleen, kidney, immune system, and within the CNS [158–162] and genetic variants of UCP2 have been associated with obesity and insulin resistance [163–165]. Interestingly, UCP2 has a dual function of protecting against ROS and supporting fatty acid oxidation [153, 154] and also presents anti-inflammatory effects by having a protective role in acute and chronic neurodegeneration and inflammatory brain diseases [166].

Hypothalamic inflammation has been connected to obesity in the past. Following HFD, pro-inflammatory proteins such as Tnfα, Il-6, and Jnk3 are upregulated in the hypothalamus in rats [167]. Prolonged exposure to HFD in rodents also leads to hypothalamic inflammation, resulting in hypothalamic leptin resistance and subsequent development of obesity due to reduced leptin effectiveness [139, 152, 168]. This inflammation in the hypothalamus has also been observed in humans and correlates with elevated levels of serum inflammatory proteins, including IL-6 and C-reactive protein (CRP) [139, 152]. Interestingly, obesity resistance has been associated with increased expression of IL-6 in the hypothalamus. It plays a role in neurogenesis within the hypothalamus. This process involves the expression of transcription factors Sox2 and Sox6, which are crucial for neurogenic transcriptional regulation [169–171]. Produced in response to exercise, primarily by muscle tissue, IL-6 has been found to mitigate memory loss in Alzheimer’s disease models [172, 173]. Moreover, exercise-induced IL-6 can reduce diet-induced inflammation and restore abnormal regulation of food intake [174, 175]. In mice fed HFD, administration of IL-6 protected against weight gain regardless of calorie intake [176].

**Fig. 4:**
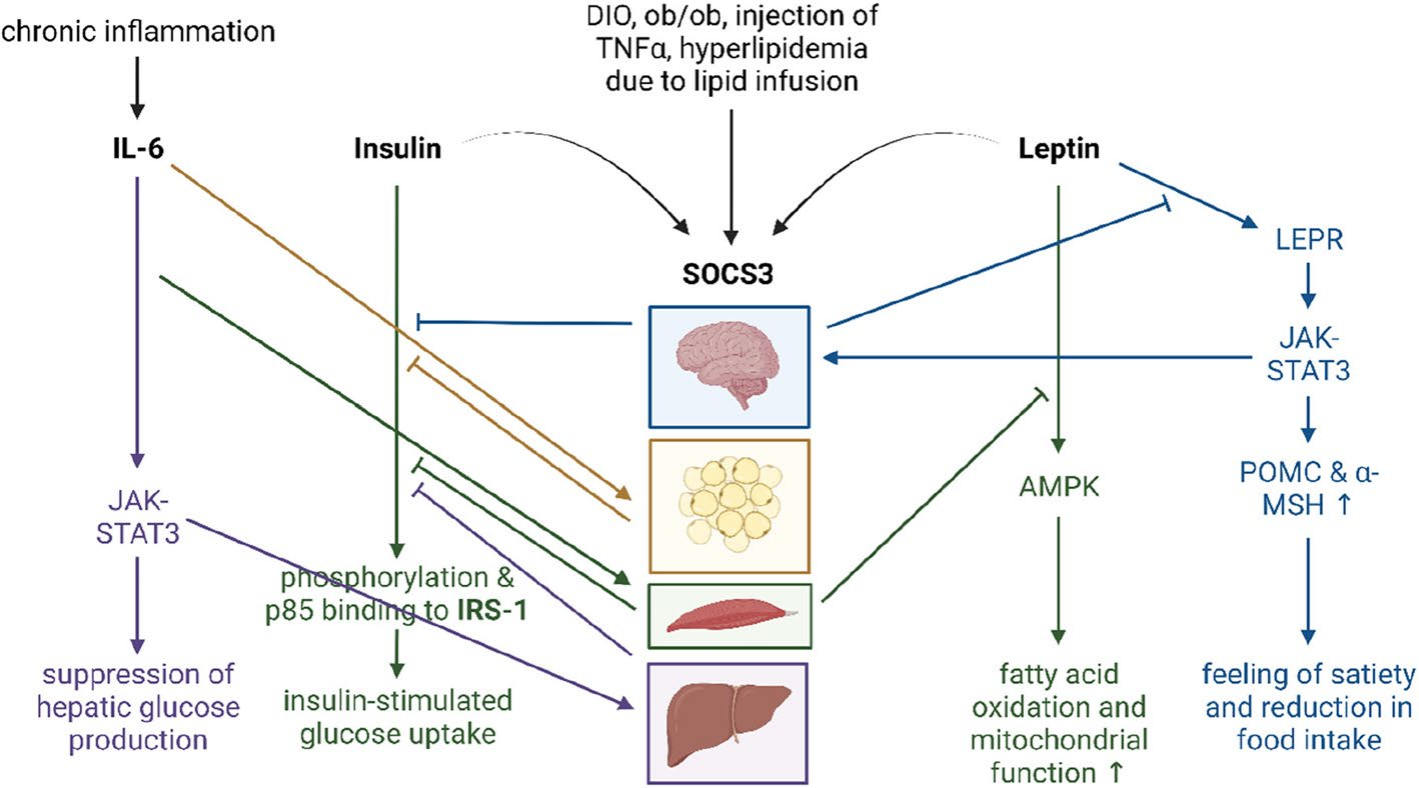
Pro-inflammatory cytokine SOCS3 has been proposed to play an important role in the development of leptin and insulin resistance in the CNS and the periphery. SOCS3 is activated and produced by DIO, injection of TNFα, or due to lipid infusion in several different organs. It is also activated upon increased levels of insulin and leptin and acts as a negative regulator for both, i.e., by inhibiting phosphorylation and p85 binding to insulin receptor substrate 1 (IRS-1), inhibiting insulin-stimulated glucose uptake or reducing the binding of leptin to the leptin-receptor in the hypothalamus, leading to orexigenic signaling. Chronic inflammation increases levels of IL-6, which in turn activates SOCS3 in the adipose tissue

Although discussed controversially as it is not known yet whether leptin resistance actually occurs in humans, its development within the CNS might be partially mediated by the activation of pro-inflammatory suppressor of cytokine signaling 3 (SOCS3) (Fig. 4). The binding of leptin to its receptor triggers the activation of the janus-kinase-signal transducer and activators of transcription-SOCS3 (JAK-STAT-SOCS3) signaling pathway [177–179]. Within this pathway, SOCS3 serves as a negative feedback regulator, exerting control over the effects of leptin and dampening the downstream activation of MC4R [179]. Accordingly, in situations where there is heightened production of leptin due to increased adipose tissue, there can also be an upregulation of SOCS3 expression. This elevated SOCS3 expression can contribute to the development of both leptin resistance and insulin resistance within the brain and peripheral tissues [179–181].

### Inflammation and liver

**Fig. 5:**
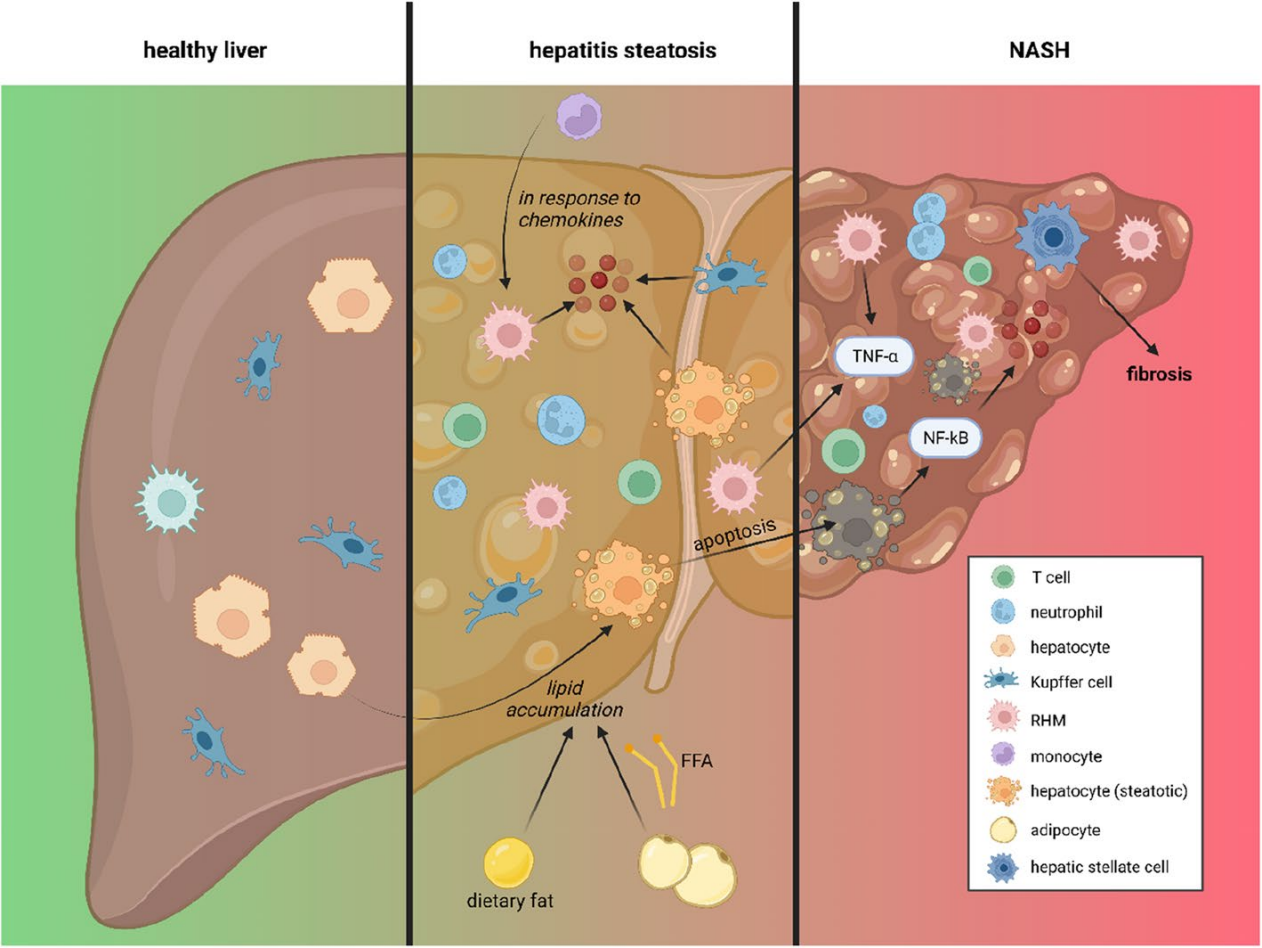
Pathophysiology from the healthy liver to NASH. In response to pathogens and FFAs pro-inflammatory cytokines are produced within the hepatic tissue by RHMs, Kupffer cells, and hepatocytes. Dietary fat due to HFD leads to lipid accumulation within the liver. Pro-inflammatory cytokines, especially an overactivation of NFκB lead to the activation of HSC, which in turn produces extracellular matrix contributing to the progression of fibrosis. Kupffer cells undergo apoptosis in a state of increased inflammation and are replaced by RHMs

**Fig. 6:**
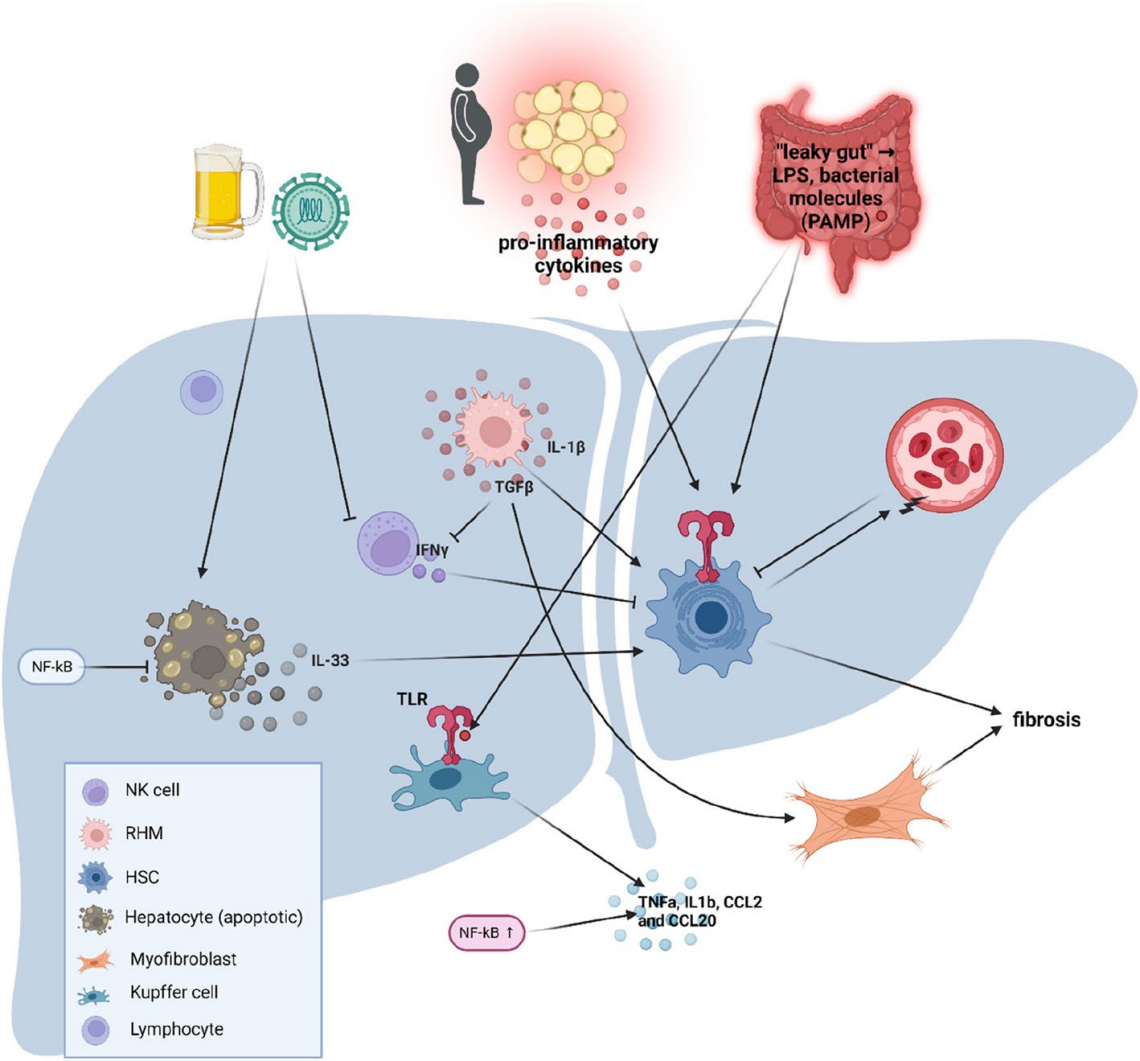
Inflammation and fibrosis in hepatic tissue. Alcohol consumption and viral infections can lead to morphologic changes in hepatocytes which proceed to undergo apoptosis. These triggers also lead to an infiltration of lymphocytes into the hepatic tissue and an inhibition of NK cells. Pro-inflammatory cytokines which are produced by adipose tissue of obese individuals can also initiate activation of HSC. Additionally, microbial changes in the gut can lead to a disturbed barrier and an increased infiltration of LPS and bacterial molecules. These bind to TLR and promote the production of pro-inflammatory cytokines via Kupffer cells and HSC activation

**Fig. 7:**
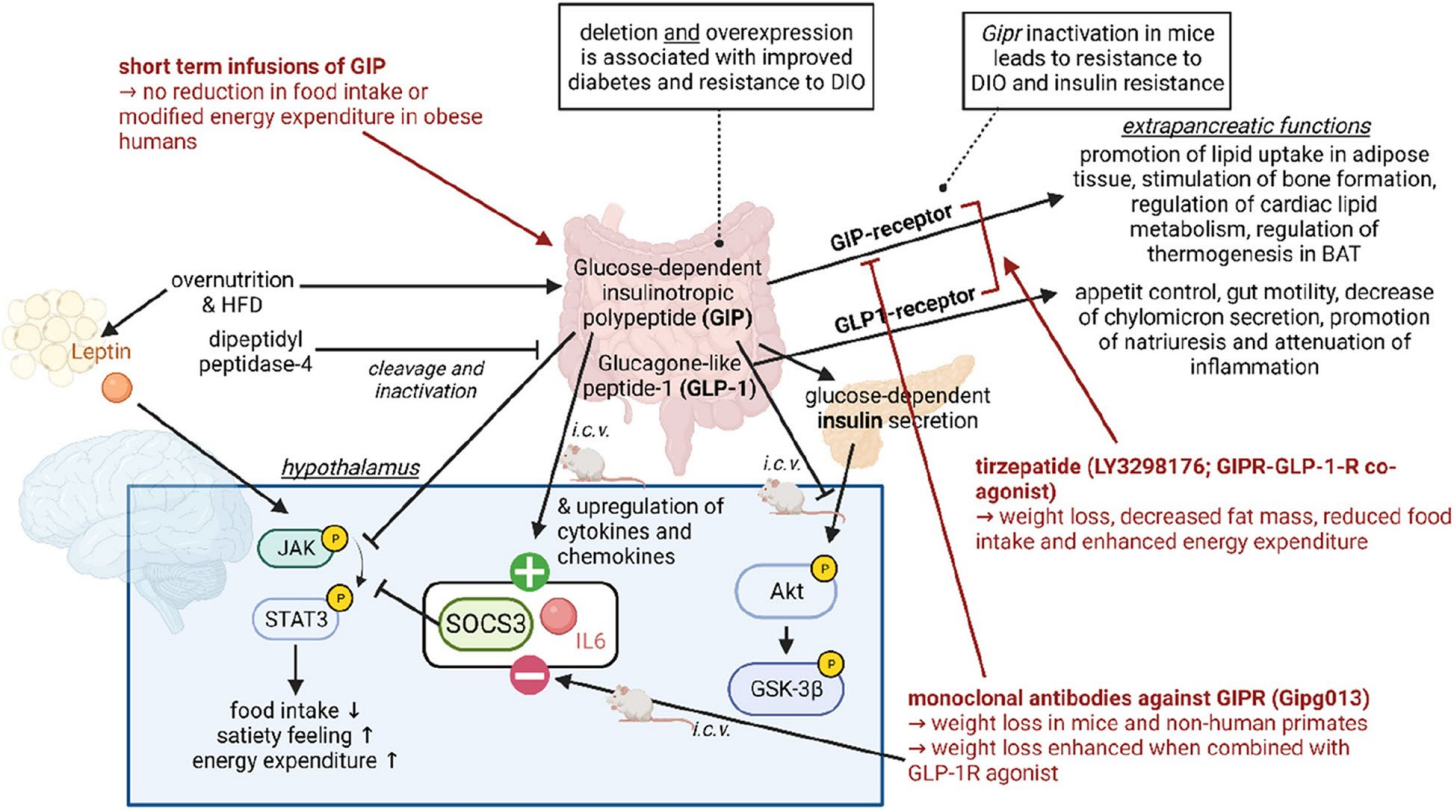
Gut hormone-derived incretins contribute to hypothalamic inflammation and modulate insulin- and leptin-resistance. Over-nutrition activates GIP production in the gut, which in turn activates glucose-dependent insulin secretion in the pancreas and also exhibits extra-pancreatic functions. Interestingly, in mice, it has been shown that deletion and overexpression of GIP is associated with improved diabetes and resistance to DIO. Centrally administered GIP leads to a reduction of JAK-STAT-activation and therefore diminishes leptin activity in the hypothalamus and upregulates SOCS3 and IL-6 in mice. Concordantly, intracerebral application of monoclonal antibodies against GIPR leads to a suppression of SOCS3 and IL-6 and induces weight loss in mice and non-human primates. This effect is enhanced when combined with GLP-1R agonist. Paradoxically, the GIPR-GLP-1R co-agonist also leads to weight loss, reduced food intake, and a decrease in fat mass. These incretins and their receptors propose immense pharmacological potential in targeting DIO and its co-morbidities

In liver disease, there is a dysregulation of the tolerogenic mechanism, leading to an excessive inflammatory response [182]. In non-alcoholic steatohepatitis (NASH), there is a persistent occurrence of apoptosis in Kupffer cells, which are subsequently replaced by monocyte-derived recruited hepatic macrophages [183] (see Figs. 5 and 6). Additionally, there is an accumulation of collagen within the liver [184, 185]. Hepatocytes become steatotic, primarily attributed to enhanced de novo lipogenesis, and exhibit a ballooned morphology characteristic of NASH [186]. Furthermore, the abnormal accumulation of triglycerides in hepatocytes, along with oxidative stress and lipid peroxidation, collectively contribute to the pathogenesis of non-alcoholic fatty liver disease (NAFLD) [187–189]. In obese individuals, adipose tissue inflammation results in the secretion of inflammatory cytokines that further promote hepatic inflammation [190]. Moreover, dysregulated hepatic lipid and cholesterol metabolism contributes to an increased production of ROS [190]. Additionally, alterations in the gut can lead to augmented infiltration of LPS, thereby inducing hepatic inflammation, hepatocyte damage, and activation of hepatic stellate cells (HSCs), which produce extracellular matrix and therefore contribute to fibrotic changes within the liver tissue [191]. Leptin also plays a significant role in the activation of HSCs [192]. Stimulation by leptin results in enhanced expression of TGFβ in Kupffer cells and upregulation of hedgehog signaling pathways that sustain the activated phenotype of HSCs [193]. Additionally, LEPR-deficient rats exhibit a protective effect against the progression of liver fibrosis induced by carbon tetrachloride (CCl4) [194]. On the other hand, adiponectin suppresses HSC activation in NASH [195, 196]. Administration of recombinant adiponectin has been shown to ameliorate hepatic steatosis and inflammation in obese mice [197]. Dual agonist of adiponectin receptors AdipoR1/AdipR2 improved NASH and fibrosis in rodents by reducing HSC activation [198]. Several potential treatment options have emerged to attenuate the progression of liver fibrosis in NASH by targeting inflammatory pathways. Among these approaches is the inhibition of cytokine-mediated processes, such as the utilization of anti-interleukin-17 (anti-IL-17) biological therapy to impede HSC stimulation [199]. Additionally, promising effects have been observed in a phase 2 clinical trial of the CCR2/5 antagonist cenicriviroc [200], which suppresses monocyte recruitment to the liver and has demonstrated a reduction of liver fibrosis in rodents [201–205]. Furthermore, the neutralization of TGFβ using fresolimumab (GC1008), a human anti-TGFβ1 monoclonal antibody, has shown successful suppression of liver fibrosis development in mouse models [206–209]. Hyperinsulinemia can directly stimulate the proliferation of HSCs and subsequently trigger the secretion of type 1 collagen [210]. In obese rats, HFD and the consequent insulin resistance were observed to elevate the expression of TGFβ1 [211]. Moreover, hyperglycemia itself can also activate HSCs [212]. Interestingly, a meta-analysis revealed that nearly all patients with T2DM also exhibit NASH [213]. Insulin inhibits lipolysis in adipocytes [214]. Upon insulin resistance in adipose tissue, elevated release of free fatty acids (FFAs) can be examined. These FFAs activate NFκB among others, and lead to lipotoxicity, which can result in lipid accumulation in the liver [190, 215]. Lipid overload potentiates oxidative stress and liver damage. Accordingly, patients with NAFLD show significantly increased serum FFA levels [216]. Additionally, overexpression of TNFα and IL-6, which occur in obese adipose tissue, can be involved in the progression of NAFLD [217, 218]. Secretion of IL-6 can elevate the expression of hepatic SOCS3, which can contribute to the development of hepatic insulin resistance [219] (see Fig. 7). Hepatic insulin resistance can also occur due to inhibitory serine phosphorylation of the insulin signaling molecules Irs1 and Irs2. This is caused by overactivation of JNK in hepatocytes in response to pro-inflammatory cytokines, ER stress and ROS [220]. Conclusively, the liver might contribute to the development of metabolic syndrome and morphologic changes in hepatic tissue are a result of increased body mass. Therefore, targeting inflammation within this organ may improve the outcome of metabolic diseases.

This is especially relevant for pediatric patients as it has been shown, that in obese adolescents insulin sensitivity and glucose tolerance as well as the risk for the development of T2DM are directly linked to liver steatosis [221, 222]. Interestingly, adolescents with NASH present with higher serum TNFα and MCP1 and lower serum adiponectin levels, thereby displaying a pro-inflammatory trend [223].

### Inflammation and incretins

Gut-derived hormones such as glucose-dependent insulinotropic polypeptide (GIP) and glucagon-like peptide 1 (GLP-1) also contribute to the development and mitigation of inflammation within the whole body including the hypothalamus (Fig. 7). Studies have demonstrated that GIP is associated with increased expression of pro-inflammatory cytokines and chemokines, while GIP infusion induces elevated levels of adipokines and pro-inflammatory cytokines in adipocytes in vitro [224–229]. Centrally administered GIP leads to an increase in pro-inflammatory cytokines and factors such as Il-6 and Socs3 in the hypothalamus in mice [230], diminishes the anorectic effects of insulin in the brain and attenuates the impact of leptin, resulting in leptin resistance [231]. Loss of GIP action is therefore associated with a better outcome in diabetes and resistance towards DIO in mice [232, 233], but contradictory a transgenic overexpression of GIP also promotes resistance to DIO and leads to a reduced fat mass in mice [234]. Genetic elimination of GIP or its receptor in mice has yielded long-term metabolic protection against diet-induced obesity and insulin resistance [224, 235–239]. In the liver, a reduction of GIP ameliorates lipid accumulation and lowers the expression of markers of inflammation [235, 240, 241]. As GIP receptor (GIPR) is also expressed by myeloid cell lines, which include monocytes and macrophages as well as bone marrow-derived T cells, *Gipr* deletion in rodents impacts hematopoiesis by decreasing the number of myeloid-progenitor cells, as well as circulating monocytes and macrophages [242, 243]. Moreover, GIPR deficiency and the application of an antagonistic GIP receptor antibody significantly diminish the levels of pro-inflammatory cytokines such as Il-6 and Socs3 in the hypothalamus in mice [230]. Acute inhibition of GIPR using neutralizing antibodies has shown the ability to substantially reduce body weight and improve obesity by enhancing the effectiveness of leptin [231, 235, 244, 245]. In humans, elevated plasma GIP levels have also been correlated with increased expression of pro-inflammatory genes in obese individuals [246]. Therefore, GIP is considered a pivotal factor in driving leptin resistance and plays a significant role in hypothalamic inflammation.

GLP-1 has also been identified as an important regulator of inflammation and metabolic diseases and has been targeted as a therapeutic opportunity to improve cardiovascular and metabolic outcomes of patients with obesity and T2DM. It is synthesized in the large and small bowel and colon as well as in the brain [247, 248]. In the pancreas, the binding of GLP-1 to its receptor (GLP-1R) stimulates insulin secretion and also increases glucose metabolism by promoting insulin synthesis [249]. Additionally, it preserves beta-cell mass through stimulation of cell proliferation and inhibition of apoptosis and is therefore improving glycemic control via chronic alterations [250–253].

The secretion of GLP-1 is induced by various factors such as inflammation, microbial metabolites, and cytokines [254–257]. Accordingly, in hospitalized patients with critical illness, plasma levels of GLP-1 correlate with the severity and survival [258, 259]. Conclusively, GLP-1 receptor agonists (GLP-1R) can reduce systemic inflammation as well as tissue inflammation in rodents independent of body weight changes [260, 261]. However, the underlying mechanism remains poorly understood [262]. In the liver, GLP-1 also reduces hepatic steatosis and inflammation and can additionally attenuate hepatocyte injury in preclinical studies with models of non-alcoholic steatohepatitis (NASH). This effect has also been shown in humans with NASH, partly independent of weight changes [263–265]. There are currently ongoing clinical studies to investigate the potential of liraglutide and semaglutide, GLP-1R agonists, to reduce hepatic inflammation in people with NASH [266, 267]. In obese patients, liraglutide administered daily for 48 weeks improved liver histology and decreased the progression of fibrosis [267]. The exact underlying mechanism remains unclear. Interestingly, GLP-1R has been detected in the endothelium, the coronary arteries, and the smooth muscle cells of the heart [268, 269]. In cardiovascular outcome trials, GLP-1RA reduced the rates of major adverse cardiovascular events (MACEs) and liraglutide administration reduced total mortality, cardiovascular death, and number of myocardial infarctions in patients [270, 271]. It has also been shown that liraglutide improved behavioral profile and induced re-myelination in a mouse model of multiple sclerosis (MS). These effects are proposed to be due to anti-inflammatory, autophagic flux activation, and inflammasome suppression [272]. In an in vivo model for experimental autoimmune encephalitis (EAE), liraglutide could ameliorate the disease score, was able to delay the disease onset and reduce demyelination and inflammation scores in the lumbar spinal cord [273]. These results suggest the anti-inflammatory effects of GLP-1R agonists in the central nervous system and a potential therapy option for patients with MS or autoimmune encephalitis.

As GLP-1R agonists like liraglutide and semaglutide have shown very promising results in clinical studies, the development of dual- and tri-agonists, which also have agonist effects on glucagon-receptors, holds immense promise to improve metabolic outcomes for people with obesity, T2DM or liver diseases [248]. The dual agonist tirzepatide is achieving tremendous weight loss in patients with T2DM, improves blood glucose levels, and reduces hepatic steatosis. Interestingly, tri-agonists also show neuroprotective effects in rodent models of Alzheimer's disease and Parkinson’s disease [241, 274–281].

## Conclusion

There has been clear evidence in vitro, in rodents and in humans, that obesity and inflammation are significantly interconnected and effect each other on several metabolic levels. Additionally, targeting inflammation in the adipose tissue or the hypothalamus introduces new possibilities to prevent diet-induced obesity as well as insulin and leptin resistance. In this review, we displayed the important interplay between gut hormones, adipose tissue, and the hypothalamus in regard to inflammation as this is an important pathomechanism in advancing therapy options for obesity. This is of strong importance for pediatric patients because the conversion from impaired glucose tolerance to the development of T2DM does not seem to be a linear process. The progression appears much faster in children and adolescents compared to adults [282]. Additionally, it has been shown that the 20-year survival rate free of liver transplant for children with NAFLD was about 80% compared to 99% in the reference population [283]. There is an urgent need for a deeper understanding of the development of comorbidities and the interplay of different organ systems, hormones, and cytokines, especially in early life stages.

## Data Availability

Not applicable.

## Abbreviations

T2DM: Type 2 diabetes mellitus
IL-6: Interleukin-6
TNFa: Tumor necrosis factor-α
LEPR: Leptin-receptor
POMC: Proopiomelanocortin
MSH: Melanocyte-stimulating hormone
MC4R: Melanocortin-4-receptor
LEP: Leptin
LPS: Lipopolysaccharide
Th: T helper cell
DSS: Dextran sulfate sodium
NK cells: Natural killer cells
INFγ: Interferon-γ
IgE: Immunoglobulin E
NO: Nitric oxide
Groα: Growth-regulated protein α
NFκB: Nuclear factor “kappa-light-chain-enhancer” of activated B cells
MC1R: Melanocortin-1-receptor
SIRS: Systemic inflammatory response syndrome
FDA: Food and Drug Administration
EMA: European Medicines Agency
CCL2: Chemokine C–C motif ligand 2
CXCL10: C-X-C motif chemokine 10
CNS: Central nervous system
UPR: Unfolded protein response pathway
TLR: Toll-like receptors
JNK1: Janus kinase 1
MCP1: Monocyte chemoattractant protein-1
CVD: Cardiovascular diseases
VCAM1: Vascular cell adhesion molecule 1
M1: Macrophage type 1
HFD: High-fat diet
ATM: Adipose tissue macrophage
DIO: Diet-induced obesity
UCP2: Uncoupling protein 2
ROS: Reactive oxygen species
CRP: C-reactive protein
SOCS3: Suppressor of cytokine signaling 3
JAK: Janus kinase
STAT: Signal transducer and activator of transcription
GIP: Glucose-dependent insulinotropic polypeptide
GLP-1: Glucagon-like peptide 1
GIPR: GIP receptor
GLP-1R: GLP-1 receptor
GLP-1RA: GLP-1 receptor agonists
NASH: Non-alcoholic steatohepatitis
MACE: Major adverse cardiovascular event
MS: Multiple sclerosis
EAE: Experimental autoimmune encephalitis
TGFβ: Transforming growth factor β
IRS-1: Insulin receptor substrate 1
RHM: Recruited hepatic macrophage
HSC: Hepatic stellate cells
NAFLD: Non-alcoholic fatty liver disease
FFA: Free fatty acid
AGE: Advanced glycation end product
